# Standardizing Rectal Suction Biopsy Preparation With a Dedicated Equipment Box: A Quality Improvement Project

**DOI:** 10.7759/cureus.116518

**Published:** 2026-09-18

**Authors:** Yasir Alkhatib

**Affiliations:** 1 Pediatric Surgery, Noah's Ark Children's Hospital for Wales, Cardiff, GBR

**Keywords:** equipment standardization, hirschsprung disease, paediatric surgery, quality improvement, rectal suction biopsy, workflow efficiency

## Abstract

Rectal suction biopsy is an established diagnostic procedure for suspected Hirschsprung disease, but reliable performance depends on having multiple pieces of equipment, consumables, specimen containers, and documentation immediately available. In our paediatric surgical clinical area, the required items were stored in different locations, resulting in avoidable searching, workflow interruptions, and the possibility of missing essential equipment during preparation. A quality improvement (QI) project was undertaken to standardize preparation through a dedicated, clearly labelled rectal suction biopsy equipment box. A procedure-specific checklist was developed, the box was placed in an agreed accessible location, and staff were oriented to its use and the restocking process. The intervention was trialled for more than one month and evaluated using an anonymous staff survey and observational feedback. Contemporaneous project documentation recorded improved immediate equipment availability, reduced time spent searching for items, improved workflow efficiency, and high staff satisfaction. Sustainability measures included a contents checklist inside the box, assigned responsibility for restocking, and a permanent storage location. The original response-level survey dataset and objective timing measurements were not available for re-analysis when this report was prepared; therefore, no percentages, p-values, or numerical effect estimates are presented. This low-cost intervention provides a reproducible framework for standardizing preparation for a specialized paediatric surgical procedure. Future cycles should prospectively record preparation time, missing-item events, restocking failures, cost, and sustained compliance.

## Introduction

Hirschsprung disease is a congenital disorder of the enteric nervous system for which diagnosis depends on representative rectal histology. Rectal suction biopsy is a minimally invasive technique in which suction is used to obtain small samples of rectal mucosa and submucosa for histopathological assessment, allowing evaluation for the absence of ganglion cells and associated diagnostic features. Because an adequate specimen must contain sufficient submucosa, reliable preparation of the biopsy device, specimen containers, consumables, and documentation is important before the procedure begins. Updated European Reference Network guidance continues to place rectal biopsy at the centre of diagnostic confirmation, with biopsy technique and specimen adequacy remaining important components of the diagnostic pathway [1]. A systematic review and meta-analysis of paediatric rectal biopsy techniques found that suction biopsy provides a high proportion of conclusive samples with a very low pooled complication rate [2].

For a focused procedure such as rectal suction biopsy, technical success is influenced not only by the biopsy device and operator but also by the reliable preparation of consumables, specimen containers, forms, and supporting equipment. Equipment standardization has been used successfully in paediatric surgery to reduce costs, reduce variation, and improve perceived efficiency. Standardized laparoscopic appendectomy equipment reduced operative supply costs without worsening operative outcomes [3], while standardization of paediatric inguinal hernia instrument sets substantially reduced tray size and processing time [4].

The local problem in our unit was operational rather than diagnostic: the equipment required for rectal suction biopsy was stored in multiple locations. Staff therefore had to collect individual items before each procedure, sometimes leaving the immediate clinical area to locate them. This introduced avoidable delays and the possibility that an item might be omitted during preparation. Similar hospital Quality Improvement (QI) work has shown that procedure-specific equipment organization can reduce the time spent gathering equipment [5,6].

The aim of this project was to improve the immediate availability of essential rectal suction biopsy equipment and reduce avoidable preparation delays by introducing a dedicated standardized equipment box. This report describes the intervention, the QI cycles, the documented staff feedback, and the sustainability strategy, with reporting informed by Standards for Quality Improvement Reporting Excellence (SQUIRE) 2.0 principles [7].

## Technical report

Local problem and aim

Before the intervention, equipment required for rectal suction biopsy was distributed across several storage locations. Staff reported time lost searching for basic items, an occasional need to leave the clinical area, and concern that essential equipment could be missed. The project aim was to improve immediate equipment availability and reduce preparation delays by creating a single standardized source for the routinely required items. A fishbone/root-cause analysis was constructed from the documented pre-intervention workflow problems to summarize the principal contributors to delay and variation (Figure 1).

**Figure 1:**
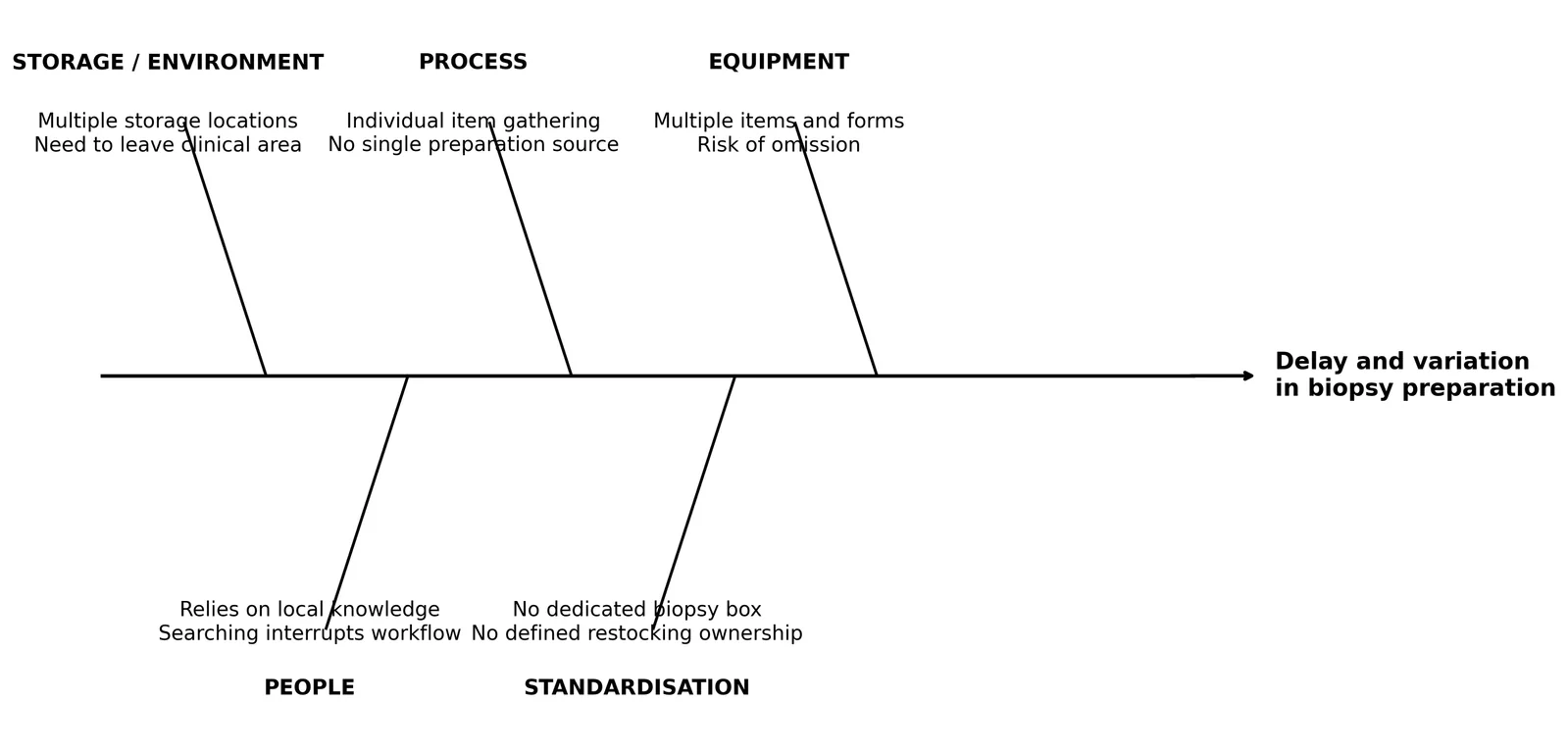
Fishbone/root-cause analysis of documented pre-intervention contributors to delays and variation in rectal suction biopsy preparation. Source: Original figure created by the author based on contemporaneous quality improvement project documentation. No quantitative baseline data were reconstructed.

Intervention design

A dedicated rectal suction biopsy box was assembled using the locally established workflow. The box was clearly labelled, stored in a designated accessible location, and accompanied by a checklist. Staff were informed of its location, use, and restocking process. The intervention did not change the indication for biopsy, the biopsy technique, or the clinical diagnostic pathway; it reorganized the preparation process around a single procedure-specific equipment source. The standardized box contents are listed in Table 1.

**Table 1:** Standard contents of the rectal suction biopsy equipment box. Source: Original table created by the author based on local quality improvement project documentation.

Category	Standardized contents
Biopsy equipment	Biopsy handpiece; pack of three capsules
Consumables	10 mL syringe; lubricating gel; gauze; needle; saline; gallipot
Specimen handling	Three white-top universal specimen containers
Documentation	Histopathology form; sterile equipment forms
Supporting items	Equipment bag; sucrose; fresh nappy

QI approach and Plan-Do-Study-Act (PDSA) cycles

The project was registered on the hospital QI portal. The original project plan identified preparation time from the decision to perform the biopsy to the start of the procedure as a process measure, staff satisfaction and missing equipment as outcome measures, and the cost of creating and maintaining the box as a balancing measure. The documented PDSA sequence is summarized in Table 2.

**Table 2:** Documented PDSA sequence. Source: Original table created by the author based on the documented PDSA activities undertaken during this quality improvement project. PDSA: Plan-Do-Study-Act.

Cycle	PDSA step	Action
Cycle 1 - Initial trial	Plan	Identify the recurring preparation problem and define the items required for a standard rectal suction biopsy.
Cycle 1 - Initial trial	Do	Create one standardized box and trial it in the clinical area for more than one month.
Cycle 1 - Initial trial	Study	Collect anonymous staff feedback and observational comments about availability, preparation, and usability.
Cycle 1 - Initial trial	Act	Continue the intervention because the documented feedback was favourable.
Cycle 2 - Sustainability	Plan	Address the risk of missing items and inconsistent restocking.
Cycle 2 - Sustainability	Do	Place a contents checklist inside the box, assign responsibility for restocking, and agree on a permanent storage location.
Cycle 2 - Sustainability	Study	Confirm that the box was incorporated into routine workflow and remained well received by staff.
Cycle 2 - Sustainability	Act	Plan a re-audit at three to six months and prospective capture of quantitative process measures.

Staff evaluation

After more than one month of box use, staff feedback was collected using an anonymous questionnaire. The questionnaire compared staff experiences before and after implementation and assessed preparation, missing equipment, perceived preparation time, workflow, and overall satisfaction. The questionnaire items are shown in Table 3.

**Table 3:** Anonymous staff feedback questionnaire used after implementation. Source: Original questionnaire developed by the author as part of this quality improvement project. SHO: Senior House Officer; ANP: Advanced Nurse Practitioner.

Item	Question	Response options
1	Role	Consultant / Registrar / SHO / ANP / Other
2	Before the box: ease of gathering all required equipment	Very difficult / Difficult / Neutral / Easy / Very easy
3	Before the box: frequency of missing or forgotten items	Always / Often / Sometimes / Rarely / Never
4	After the box: ease of preparing for rectal suction biopsy	Very difficult / Difficult / Neutral / Easy / Very easy
5	The box reduced preparation time	Strongly disagree / Disagree / Neutral / Agree / Strongly agree
6	The box reduced the risk of missing essential equipment	Strongly disagree / Disagree / Neutral / Agree / Strongly agree
7	The box improved patient safety and workflow	Strongly disagree / Disagree / Neutral / Agree / Strongly agree
8	Overall satisfaction with the standardized box	Very dissatisfied / Dissatisfied / Neutral / Satisfied / Very satisfied
9	Suggestions for improvement	Free text

Documented outcomes

Contemporaneous QI records documented improved immediate availability of equipment, reduced time spent searching for items, a positive effect on workflow efficiency, and high staff satisfaction after implementation. These findings supported continued use of the box and the move to a formal sustainability arrangement. The documented outcomes are summarized in Table 4.

**Table 4:** Outcomes supported by the available project documentation. Source: Original table created by the author based on contemporaneous quality improvement project documentation. QI: Quality improvement.

Outcome domain	Recorded finding	Evidence status
Immediate availability of required equipment	Improved after introduction of the box	Documented qualitative QI finding
Time spent searching for equipment	Reduced after introduction of the box	Documented qualitative QI finding
Workflow efficiency	Positive impact reported	Documented qualitative QI finding
Staff satisfaction	High	Documented qualitative QI finding
Frequency of missing equipment	Included in the evaluation, but response-level results were unavailable for re-analysis	Not quantified
Preparation time from decision to procedure start	Planned process measure, but objective measurements were unavailable for re-analysis	Not quantified
Patient safety outcome	Not objectively measured	No direct safety effect claimed

The individual response-level survey data and objective timing measurements were not available when this manuscript was prepared. Accordingly, percentages, p-values, confidence intervals, and numerical estimates of time saved have not been reconstructed from memory or invented. The results should therefore be interpreted as a descriptive implementation evaluation rather than a quantified effectiveness study.

Governance and sustainability

The project was registered on the hospital QI portal and evaluated a local service-process intervention using anonymous staff feedback. No patient-identifiable data were collected for this evaluation. Sustainability measures consisted of a contents checklist inside the box, assigned responsibility for restocking, and an agreed permanent storage location. A re-audit at three to six months was planned to evaluate sustained performance.

## Discussion

This project addressed a recurrent systems problem by reorganizing the equipment required for a specialized paediatric surgical procedure into a single standardized preparation source. The intervention was deliberately simple: it did not change clinical decision-making or biopsy technique but reduced workflow variation around preparation.

The rationale is supported by published paediatric surgical work. Avansino JR et al. demonstrated that standardization of operative equipment for laparoscopic appendectomy reduced supply costs by approximately 20% without adversely affecting operative time, length of stay, or intraoperative complications [3]. Koyle MA et al. used improvement-science methodology to reduce and standardize instrumentation for paediatric inguinal hernia repair, shortening instrument-processing time and receiving favourable staff feedback regarding efficiency and quality [4]. These studies support standardization as a legitimate target for service improvement in paediatric surgery.

The intervention also aligns closely with QI work outside the operating theatre. Karapinar Y et al. described procedure-specific trays and treatment-room standardization to improve equipment-gathering efficiency [5]. More recently, Glover L et al. reported a QI project in which reorganizing venepuncture and cannulation equipment markedly reduced average gathering times after successive PDSA cycles [6]. The common principle is that a predictable equipment layout reduces unnecessary searching and cognitive load.

The main strengths of the present project are its low cost, direct relevance to day-to-day paediatric surgical practice, ease of replication, and explicit sustainability plan. A procedure-specific contents list allows the intervention to be reproduced or adapted in other units. The project also involved both medical and nursing users through anonymous feedback rather than relying only on the opinion of the project lead.

The principal limitation is the absence of retained raw outcome data. Although the contemporaneous project documentation records improved equipment availability, reduced searching, better workflow, and high satisfaction, the magnitude of change cannot now be calculated. The planned process measure of preparation time was not available for analysis. This prevents statistical testing and limits causal inference. In addition, the post-intervention questionnaire asked staff to compare their current experience with the pre-intervention state, introducing recall and response bias. Future survey responses should therefore be collected immediately after individual rectal suction biopsy procedures rather than relying on retrospective comparison.

A second limitation is that patient-level outcomes were not measured. It would therefore be inappropriate to claim that the intervention directly improved patient safety, diagnostic yield, or complication rates. The defensible conclusion is that the intervention standardized preparation and reduced a recognized source of workflow friction. The next cycle should prospectively record preparation time for each procedure, missing-item events, occasions when staff leave the clinical area to retrieve equipment, restocking failures, maintenance costs, and staff satisfaction collected immediately after the procedure (Appendix 1). A run chart across consecutive biopsies would allow the effect and sustainability of the intervention to be quantified more convincingly.

SQUIRE 2.0 emphasizes transparent reporting of context, intervention, study of the intervention, and limitations [7]. In keeping with that approach, missing quantitative source data are explicitly reported rather than reconstructed. This strengthens research integrity even though it reduces the amount of numerical analysis that can be presented.

## Conclusions

A dedicated rectal suction biopsy equipment box provided a simple and reproducible method for standardizing preparation for a specialized paediatric surgical procedure. The intervention brought routinely required equipment into one accessible location and was associated, according to contemporaneous QI documentation, with improved perceived equipment availability, less time spent searching, better workflow efficiency, and high staff satisfaction. These outcome findings were derived primarily from subjective, qualitative staff feedback rather than prospectively measured quantitative endpoints. A contents checklist, assigned responsibility for restocking, and a permanent storage location were introduced to support sustained use.

Because the original response-level survey data and objective timing measurements are unavailable, the magnitude of improvement cannot be quantified, and a direct patient-safety benefit cannot be claimed. The present project therefore demonstrates feasibility, acceptability, and workflow standardization rather than a quantified effect size. Prospective procedure-level measurement during the next QI cycle would strengthen the evidence for effectiveness, cost, and sustainability.

## Appendices

Appendix 1 

**Table 5:** Prospective data-collection sheet for the next PDSA/re-audit cycle. The following minimum dataset is proposed for the next QI cycle. Completing it prospectively for consecutive biopsies would provide the quantitative evidence currently missing from the first cycle. PDSA: Plan-Do-Study-Act; Y/N: Yes/no; Min: Minutes; QI: Quality improvement.

Case	Date	Preparation time (min)	Missing item? Y/N	Left clinical area? Y/N	Restocking failure? Y/N	Approximate cost (£)
1						
2						
3						
4						
5						
6						
7						
8						
9						
10						
